# Impact of PpSpi1, a glycosylphosphatidylinositol-anchored cell wall glycoprotein, on cell wall defects of N-glycosylation-engineered *Pichia pastoris*

**DOI:** 10.1128/mbio.00617-23

**Published:** 2023-08-22

**Authors:** Quanchao Zhu, Zuyuan Jia, Yuchao Song, Weiwang Dou, Daniel Henry Scharf, Xiaodan Wu, Zhihao Xu, Wenjun Guan

**Affiliations:** 1 The Fourth Affiliated Hospital, Zhejiang University School of Medicine, Hangzhou, China; 2 China Zhejiang Provincial Key Laboratory for Microbial Biochemistry and Metabolic Engineering, Hangzhou, China; 3 Analysis Center of Agrobiology and Environmental Science of Zhejiang University, Hangzhou, China; University of Wisconsin-Madison, Madison, Wisconsin, USA

**Keywords:** N-glycosylation, humanization, cell wall, glycoprotein

## Abstract

In order to produce therapeutic glycoproteins modified with human-like N-glycan structures, much progress has been achieved toward the humanization of N-glycosylation pathways in yeasts. In this study, a *Pichia pastoris* mutant Glyco4, which has a humanized N-glycosylation pathway and could successfully generate the human-like N-glycans, was carefully characterized. Glyco4 displays a significant growth delay and cell wall defects. Comparative transcriptomics reveals that manipulating the N-glycosylation pathway could notably affect the expression pattern of numerous biological pathways in Glyco4. Among the differentially expressed genes, the down-regulation of Pp*SPI1* was proven to be the main cause of the cell wall defects in Glyco4. Deletion of Pp*SPI1* in *P. pastoris* GS115 strain presented growth delay and weaker resistance to cell wall/membrane perturbing agents. PpSpi1 was shown to be a glycosylphosphatidylinositol-anchored cell wall glycoprotein and involved in the formation of the mannoprotein layer on the outer surface of cell wall. Overexpressing Pp*SPI1* in Glyco4 could partially recover the cell wall defects and also improve its resistance to cell wall perturbing agents and osmotic stress. Thus, overexpression of PpSpi1 is a useful strategy to facilitate *P. pastoris* industrial applications in the manufacture of human glycoproteins.

**IMPORTANCE:**

Engineering of biological pathways in various microorganisms is a promising direction for biotechnology. Since the existing microbial cells have evolved over a long period of time, any artificial engineering may cause some unexpected and harmful effects on them. Systematically studying and evaluating these engineered strains are very important and necessary. In order to produce therapeutic proteins with human-like N-glycan structures, much progress has been achieved toward the humanization of N-glycosylation pathways in yeasts. The properties of a *P. pastoris* strain with humanized N-glycosylation machinery were carefully evaluated in this study. Our work has identified a key glycoprotein (PpSpi1) responsible for the poor growth and morphological defects of this glycoengineered strain. Overexpression of PpSpi1 could significantly rescue the growth defect of the glycoengineered *P. pastoris* and facilitate its future industrial applications.

## INTRODUCTION

N-Glycosylation is one of the most common post-translational modifications in eukaryotic cells (1). It is crucial for protein stability (2), activity, and function (3). Currently, most of the human glycoproteins are produced in the Chinese hamster ovary (CHO) cells (4) which could generate appropriate protein N-glycans similar to those of humans. However, the CHO cells have several non-ignorable shortcomings, such as slower growth rate, low productivity, low cell density, and susceptibility to viral infection (5). Thus, several alternative eukaryotic and prokaryotic expression systems, such as *Saccharomyces cerevisiae*, *Pichia pastoris* (also known as *Komagaetella phaffii*), and *Escherichia coli* (6)*,* have been explored for the efficient production of human glycoproteins.

Since structure of the N-glycans of other eukaryotic hosts is different from those of mammalian cells, and almost all prokaryotic cells cannot undergo N-glycosylation, a great deal of research has focused on how to humanize the N-glycosylation pathway of heterologous protein expression systems. For example, N-glycosylation of *P. pastoris* or *S. cerevisiae* is of the high-mannose type, which results in a short half-life of protein *in vivo* and might be immunogenic to humans (7). To humanize the yeast N-glycosylation pathways, the deletions of endogenous N-glycosylation genes *OCH1*/*MNN4/MNN1* in *S. cerevisiae* (8) and *OCH1*/*MNN4B*/*PNO1*/*BMT2* in *P. pastoris* were performed to achieve a more homogenous Man_8_GlcNAc_2_ oligosaccharide (9). The exogenous genes *MNS1*/*GNT1*/*MNS2*/*GNT2*/*UGE1*/*GALT* were subsequently expressed in *P. pastoris* to form the human G2 type (Gal_2_GlcNAc_2_Man_3_GlcNAc_2_) N-glycan. Finally, several human sialic acid modification-related genes were integrated into yeast chromosomes in order to coat the sialic acid groups at the end of N-glycans (9, 10).

In addition, the properties of glycoproteins produced by the glycoengineered expression systems were also carefully evaluated. For example, monoclonal antibodies produced by the humanized *P. pastoris* had been confirmed to exhibit similar clearance, steady-state volume of distribution, and half-life in wild-type mice or Fcγr−/− mice as those produced by the CHO cells (11). The immunoglobulins produced by glycoengineered *Arabidopsis thaliana* exhibited no difference in electrophoretic mobility and enzyme-linked immunosorbent assays compared to that produced by the CHO cells (12). These results indicate that humanized expression platforms have huge potential application prospects.

Although humanized N-glycosylation has been initially realized in several genera, the derived problems such as genetic instability (13), N-glycan heterogeneity (14), and growth defects of the mutants (15, 16) are still not solved, which will greatly hinder their future applications. For example, in *P. pastoris,* the disruption of Och1 (α-1,6-mannosyltransferase) which initially elongates the high-mannose N-glycan in Golgi resulted in growth delay, higher sensitivity to elevated temperature, formation of cellular agglomerates, and cell wall rearrangement (15, 17, 18). In order to address these deficiencies, several strategies were adopted, such as long-term environmental adaption (19), overexpression of *RHO1* which encodes a small GTPase to partially recover the growth defect, and strengthen the cell wall in the *S. cerevisiae* Δ*alg3*Δ*och1* strain (20). However, the results of these efforts have been less than satisfactory. In fact, so far, no human glycoproteins produced in the humanized platforms have been successfully brought to market.

In this study, a *P. pastoris* mutant Glyco4, which had undergone the humanization of the N-glycosylation pathway and could successfully generate glycoproteins modified with human-like N-glycans, was constructed. Glyco4 showed defects in growth and morphology, changes in the cell wall integrity (CWI), and increased susceptibility to cell wall perturbing agents. An uncharacterized GPI-anchored cell wall glycoprotein PpSpi1 was found to play a critical role in maintaining cell wall integrity of *P. pastoris*. The down-regulation of Pp*SPI1* in Glyco4 is the main cause of cell wall defects and poor growth. Overexpressing Pp*SPI1* in Glyco4 could partially rescue the cell wall and growth defects, which provides a useful strategy for further optimization of the humanized *P. pastoris* strains.

## RESULTS

### Humanization of N-glycosylation pathway impairs cell morphology of *P. pastoris*

**Fig 1:**
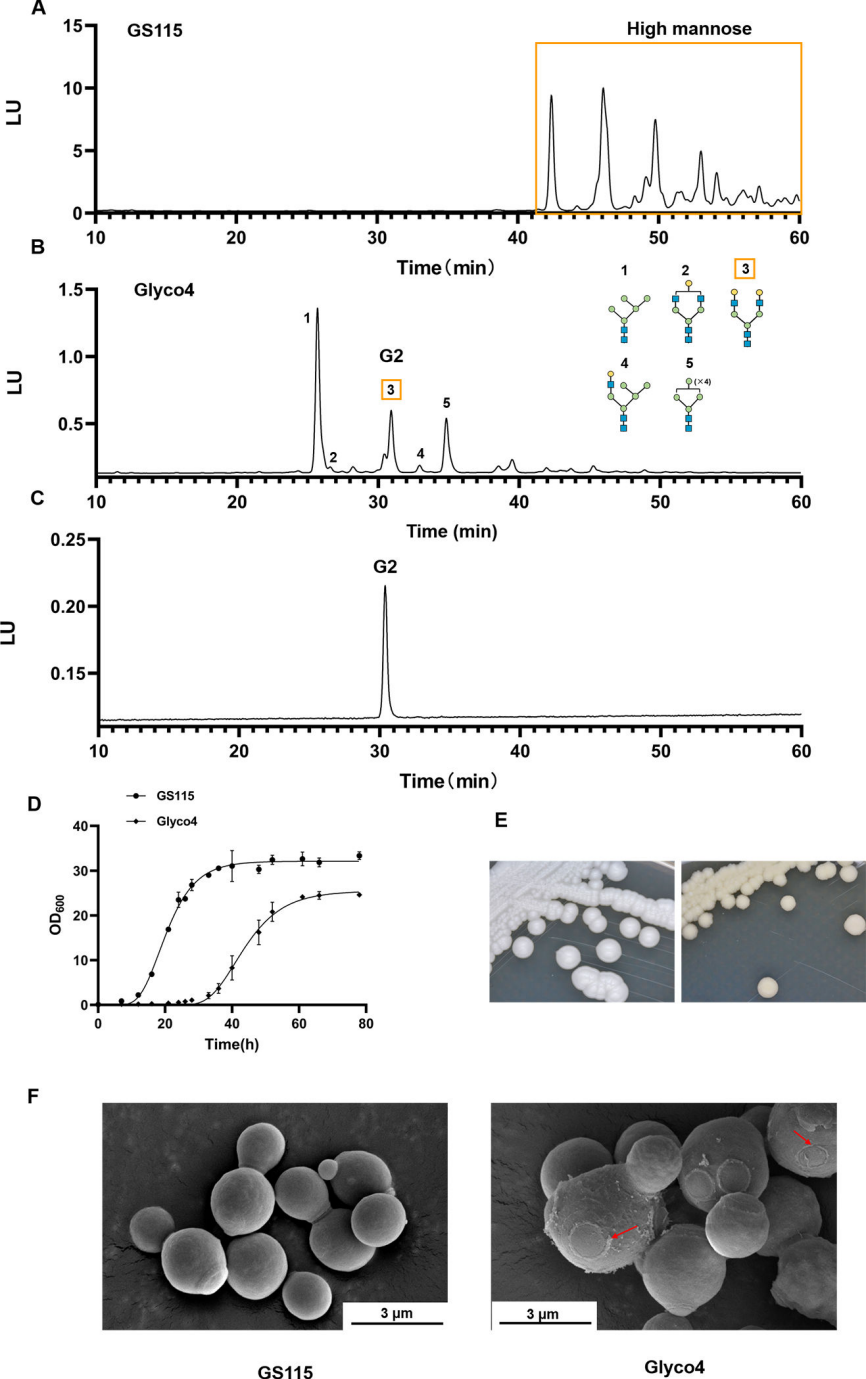
Comparison of the GS115 WT and Glyco4 strains. (**A and B**) are FLD-HPLC chromatogram N-glycans generated by the GS115 WT and Glyco4 strains. (**C**) The standard of G2 N-glycan. (**D**) The growth curves of GS115 WT and Glyco4 strains which were cultivated in 50 mL yeast extract-peptone-dextrose (YPD) broth, at 30°C, 220 rpm. (**E**) The lawn morphology of GS115 WT and Glyco4 strains on YPD agar plate, at 30°C, 48 h. (**F**) SEM observations of the GS115 WT and Glyco4 cells, 10,000×. The yeast cells were cultivated in 50 mL YPD broth, at 30°C, 220 rpm.

In this study, a humanized N-glycosylation mutant Glyco4 was constructed by integrating eight exogenous genes. The genes encording UDP-N-acetylglucosamine transporter (*Kluyveromyces lactis*), β-1,2-N-acetylglucosaminyltransferase I (*Homo sapiens*), β-1,2-N-acetylglucosaminyltransferase II (*Rattus norvegicus*), α-1,3/6-Mannosidase (*Drosophila melanogaster*), UDP-glucose 4-epimerase (*Schizosaccharomyces pombe*), UDP-galactose transporter (*D. melanogaster*), and β-1,4-galactosyltransferase (*H. sapiens*) were introduced into a *P. pastoris* GS115 mutant lacking *OCH1* (*PAS_chr1-3_0251*), *BMT2* (*PAS_chr4_0450*)*, MNN4-3* (*PAS_chr2-1_0706*)*,* and *PNO1* (*PAS_chr1-4_0410*) according to the previous report (9). As shown in Fig. 1A through C; Fig. S1, Glyco4 obviously reduced the production of high-mannose N-glycans and successfully generated the G2 type N-glycan. In liquid medium, the Glyco4 exhibited growth delay, smaller final OD_600_ value (Fig. 1D), and weaker flocculating ability (Fig. S2A) compared to the GS115 WT strain. The growth phenotype of Glyco4 on the solid medium looked less moist and smooth, also more yellow in comparison to the GS115 WT strain (Fig. 1E). Scanning electron microscopy (SEM) showed that in contrast to the GS115 WT strain with well-defined shape and smooth cell surface, Glyco4 exhibited defective bud scars, a highly heterogeneous and rougher cell surface (Fig. 1F). The results reveal that the humanization of N-glycosylation pathway leads to morphological defects and poor growth in *P. pastoris*.

### Engineering of the N-glycosylation pathway significantly affects the transcriptional profile of *P. pastoris*

**Fig 2:**
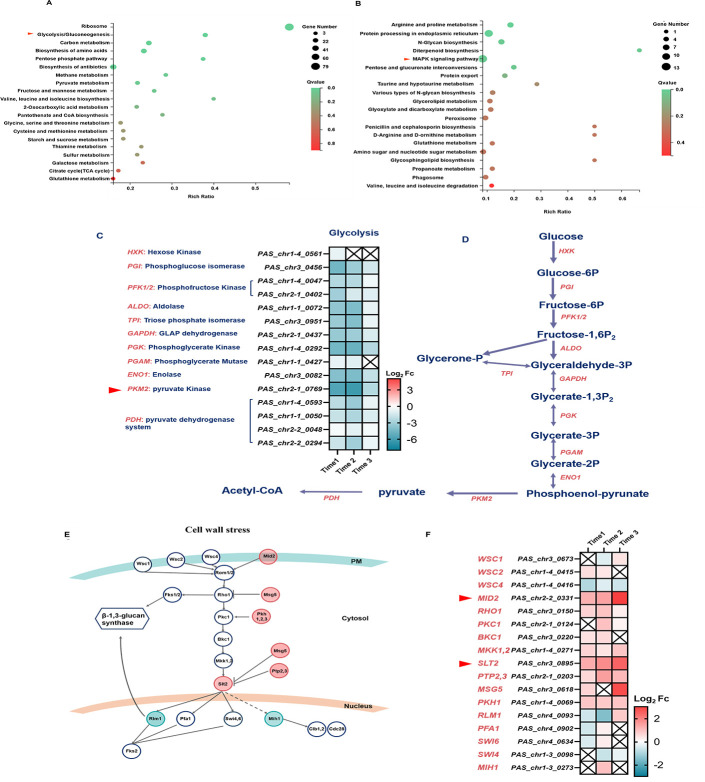
Comparative transcriptomic analysis of the GS115 WT and Glyco4 strains. The GS115 and Glyco4 strains were cultivated in 50 mL YPD broth, at 30°C, 220 rpm. The initial inoculation concentration was OD_600_ = 0.1, sampling at the lag phase, log phase, or stationary, respectively. (**A**) KEGG pathway enrichment of the down-regulated genes at sampling timepoint 1. The red triangle indicates the glycolytic pathway. (**B**) KEGG pathway enrichment of the up-regulated genes at sampling timepoint 1. The red triangle indicates the MAPK signaling pathway. *Q* value <0.05 as obviously enriched. (**C**) Fold change in the expression levels of genes involved in the glycolytic pathway. The crossed boxes indicate that the *Q*-value of log_2_Fc is greater than 0.05. (**D**) Schematic diagram of the glycolytic pathway. (**E**) Schematic diagram of the CWI pathway. Out of three sampling timepoints, the genes up-regulated (log_2_Fc >1) at least once are marked with the red circles, and the genes down-regulated (log_2_Fc <−1) at least once are marked with the green circles. (**F**) Fold change in the expression levels of genes involved in the CWI pathway, the red triangles indicate the cell wall stress sensor gene *MID2* and the key kinase gene *SLT2*.

In order to investigate how the humanization of N-glycosylation pathway impairs the growth status and cell morphology of *P. pastoris*, RNA-seq for the GS115 WT strain and Glyco4 was performed (Fig. S2B). Compared to the GS115 WT strain, the expression levels of 699, 1175, and 834 genes were significantly altered in Glyco4 at three sampling timepoints (Fig. S2C; Table S1). Based on KEGG (Kyoto encyclopedia of genes and genome) (21) pathway analysis, most of the down-regulated genes were enriched in primary metabolism-related pathways such as ribosome, carbon metabolism, glycolysis, and biosynthesis of amino acids (Fig. 2A). Notably, the expression levels of almost all genes in the glycolytic pathway were significantly lower in Glyco4. For example, the expression level of pyruvate kinase gene *PKM2*, which encodes a rate-limiting enzyme in glycolysis, was down-regulated over threefold (Fig. 2C and D). The down-regulation of the glycolytic pathway might be one of the important reasons for growth delay of Glyco4. On the other hand, many of the up-regulated genes were enriched in pathways such as protein processing in endoplasmic reticulum and MAPK (mitogen-activated protein kinase) signaling (Fig. 2B). The activation of former pathway was speculated to be mainly caused by the high expression of eight exogenous N-glycosylation-related genes in Glyco4 (Fig. S2D). Among the genes related to the CWI pathway, the stress sensor gene *MID2* (22) and the key kinase gene *SLT2* (also named as *PIM1*) (23) were significantly up-regulated in Glyco4 (Fig. 2E and F). Since both Mid2 and Slt2 play the important roles in the CWI pathway, these results imply that the humanization of N-glycosylation pathway in *P. pastoris* impairs the cell wall integrity and subsequently affects the cell growth and morphology.

### Engineering of the N-glycosylation pathway impairs the cell wall integrity of *P. pastoris*

**Fig 3:**
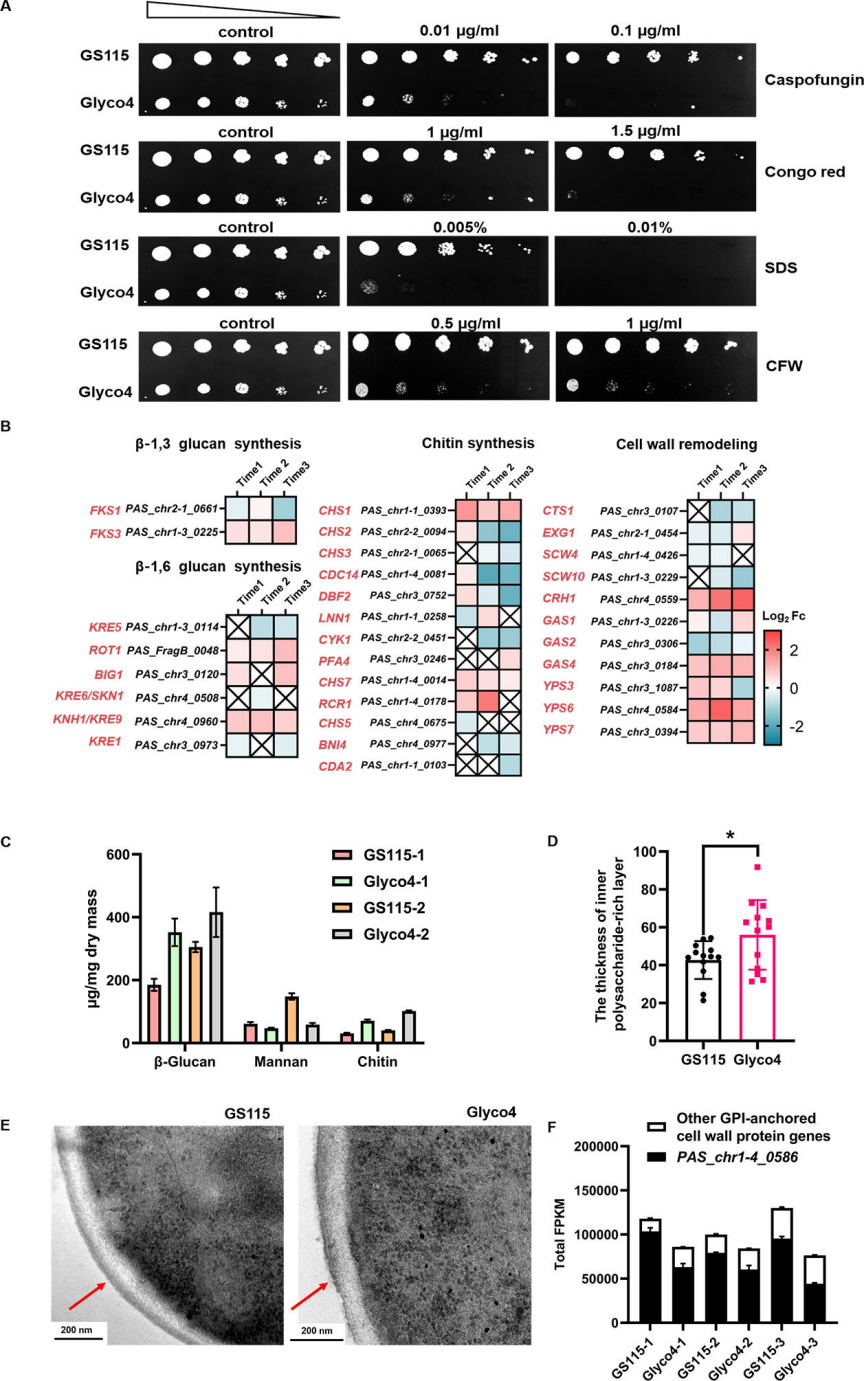
The effects on cell wall after humanizing the N-glycosylation pathway in *P. pastoris*. (**A**) Spot plate assay for caspofungin, Congo red, SDS, and CFW resistance. The same control image was used as a representative image. The yeast cells were incubated at 30°C for 48 h for observation. (**B**) Transcriptomic analysis of the cell wall synthesis and cell wall remodeling-related genes. The fold change (Fc) is the normalized log_2_ ratio of DEseq2 (DEseq2_Glyco4_/DEseq2_GS115_). The crossed boxes indicate that the *Q*-value of log_2_Fc is greater than 0.05. (**C**) Quantitative analysis of the cell wall components at logarithmic phase 1 and 2 (OD_600_ = 3 and 20, respectively). The value was calculated as micrograms of polysaccharide per milligram of cell wall dry weight. (**D**) The thickness of inner polysaccharide-rich layer of cell wall, the estimation was performed by ImageJ software (25). (**E**) Transmission electron microscopy (TEM) images of the GS115 and Glyco4 cells. The red arrows indicate the mannoprotein layer on cell wall surface. (**F**) The expression level of *PAS_Chr1-4_0586* accounted for the total FPKM value of the forty-one predicted GPI-anchored cell wall protein genes.

The cell wall of *P. pastoris* is mainly composed of mannoproteins, β-glucan (including β-1,3/6-glucan) and chitin. These components cross-link with each other to form complex and dynamic three-dimensional structures in order to protect the cell from environmental stress (24). To study the cell wall defects in Glyco4, we tested the sensitivity of GS115 WT and Glyco4 strains to four cell wall/membrane perturbing agents, including caspofungin, congo red, calcofluor white (CFW), and sodium dodecyl sulfate (SDS), which could block the synthesis of β-1,3 glucan, chitin, and damage the cell membrane, respectively. As shown, Glyco4 is more sensitive to all the above agents compared to the GS115 WT strain (Fig. 3A). This provides additional evidence that humanized N-glycosylation could cause cell wall defects in *P. pastoris*.

To further study whether the cell wall structure is disturbed in Glyco4, we evaluated the expression levels of cell wall biogenesis and remodeling genes based on the transcriptomic data. As shown in Fig. 3B, the transcriptional levels of many β-1,3 and β-1,6 glucan synthesis-related genes were up-regulated in the different growth phases. Several genes involved in chitin synthesis and cell wall remodeling also exhibited increased expression levels in Glyco4. The cell wall polysaccharides analysis showed that the contents of β-glucan and chitin in Glyco4 were increased nearly onefold, but the content of mannan was only half of that in the GS115 WT strain (Fig. 3C). In addition, the thickness of inner polysaccharide-rich layer (chitin and β-glucan) of the cell wall was increased in Glyco4 compared to that of the GS115 WT strain (Fig. 3D).

In order to find out what causes the decrease of mannan content in the cell wall of Glyco4, the cell wall structure was observed with transmission electron microscopy (TEM). The GS115 WT strain showed a uniform cellular architecture with well-defined layers of mannoprotein and β-glucan, while the Glyco4 cells exhibited an irregular edge of the cell wall and a thinner outer layer of mannoproteins (Fig. 3E), implying a reduction in cell wall mannoproteins. According to a previous report (26), 50 genes were predicted to encode cell wall GPI proteins in *P. pastoris*, and 41 of them were transcribed (FPKM value >10) in both of the GS115 WT and Glyco4 strains in this study (Table S2). Among the 41 genes, the expression levels of most flocculation-related genes were down-regulated, which is consistent with the lower flocculation ability of Glyco4 (Fig. S2A). More importantly, a super-high-expressed gene *PAS_Chr1-4_0586*, whose FPKM value accounted for more than 50% of the total FPKM value of 41 genes in both the GS115 WT and Glyco4 strains (Fig. 3F), was significantly down-regulated in Glyco4 compared to the GS115 WT strain (Table S1).

### PpSpi1 is a GPI-anchored cell wall protein

**Fig 4:**
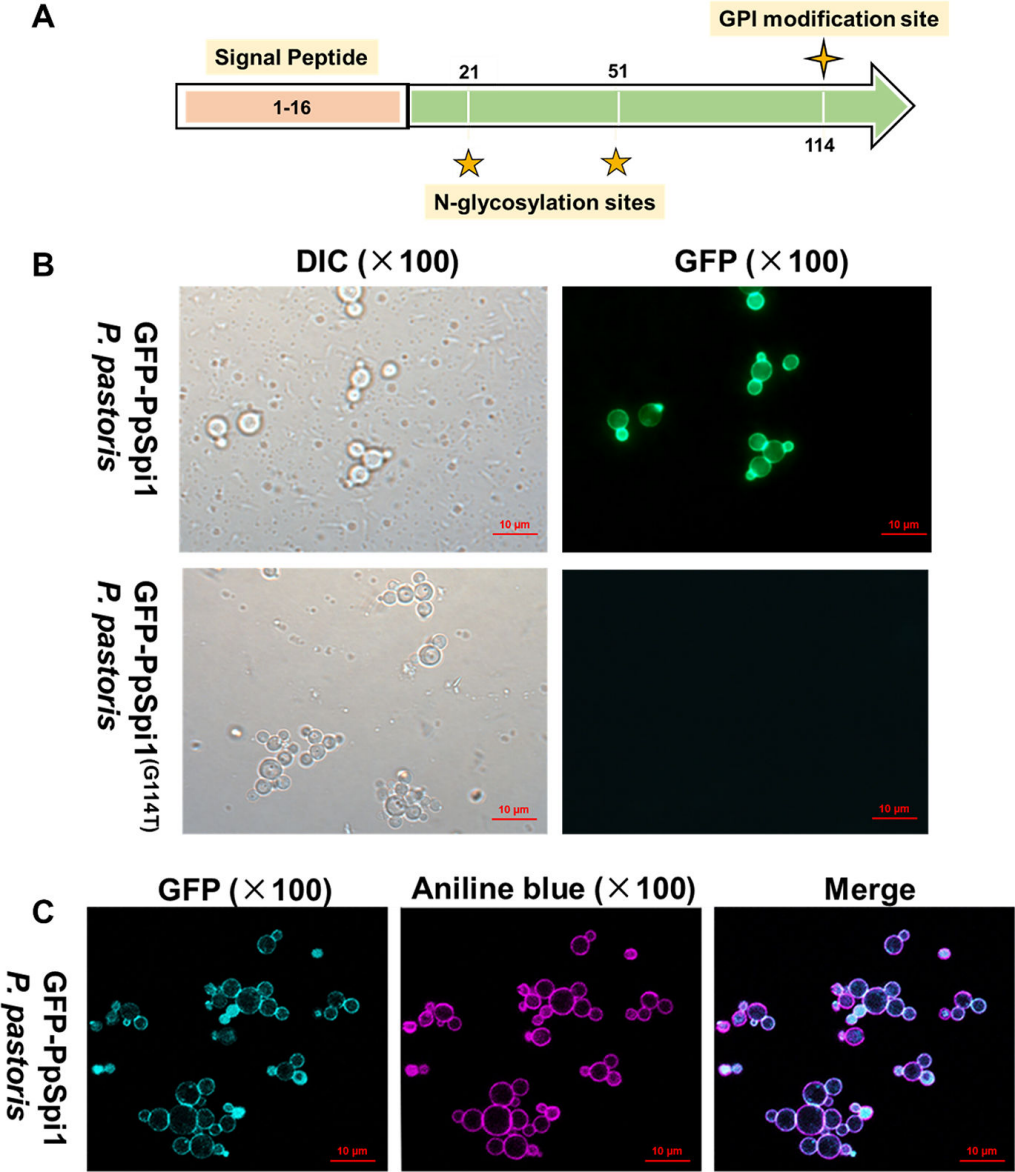
The sequence features and cell wall localization of PpSpi1. (**A**) The sequence features of PpSpi1. (**B**) Localization of GFP-PpSpi1 and GFP-PpSpi1^G114T^. (**C**) Co-localization of aniline blue staining and GFP-PpSpi1. EGFP: Ex/EM 488 nm/509 nm, detection wavelength 450–700 nm, AF405: Ex/EM 401 nm/422 nm, detection wavelength 400–496 nm. In order to enhance the contrast of the colors, the green fluorescence of GFP was converted to aquamarine blue, and the blue fluorescence of aniline blue was converted to purple by using ZEN2 software. Adobe Photoshop (PS) CC 2018 was used for image editing.

The function of the highly expressed *PAS_Chr1-4_0586* gene in *P. pastoris* was unknown. The corresponding protein was predicted to contain an N-terminal signal peptide sequence of 16-residue in length, a C-terminal GPI modification site located at glycine residue 114, and two predicted N-glycosylation sites at asparagine residue 21 and 51 (Fig. 4A). As its amino acid sequence shares 49.1% identity with ScSpi1 of *S. cerevisiae* (Fig. S3), *PAS_Chr1-4_0586* is named Pp*SPI1* in this study.

In order to confirm that PpSpi1 is a GPI-anchored cell wall protein, a vector carrying *GFP*-Pp*SPI1* or *GFP*-Pp*SPI1* ^(G114T)^ mutant was, respectively, transformed into *P. pastoris* for expression. The strain expressing GFP-PpSpi1 showed localized green fluorescence at the cell wall (Fig. 4B). But, the GPI site mutated strain *GFP*-Pp*SPI1* ^(G114T)^ displayed no fluorescence signal. The co-localization of aniline blue (a dye specifically labeling β-1,3-glucan) staining and GFP-PpSpi1 (Fig. 4C) further confirmed that PpSpi1 is a GPI-anchored cell wall protein.

### PpSpi1 is important for cell wall integrity

**Fig 5:**
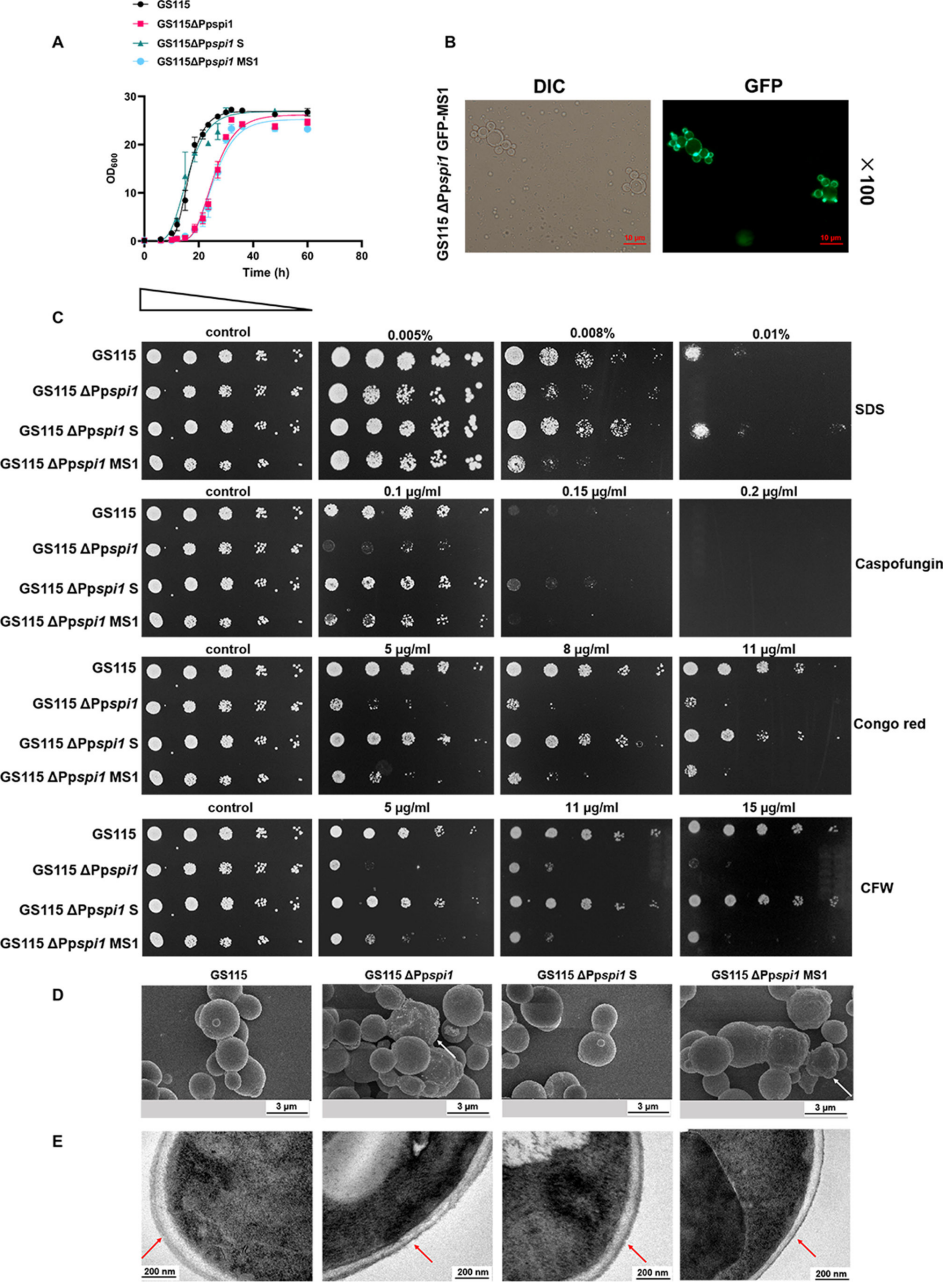
Functional study of PpSpi1. (**A**) The growth curves of GS115 WT, GS115 ΔPp*spi1*, GS115 ΔPp*spi1* S, and GS115 ΔPp*spi1* MS1 strains. The strains were cultivated in 50 mL YPD broth, at 30°C, 220 rpm. (**B**) Localization of GFP-PpSpi1^(N21D, N51D)^. (**C**) Spot plate assay for caspofungin, Congo red, SDS, and CFW resistance of the GS115 WT, GS115 ΔPp*spi1*, GS115 ΔPp*spi1* S, and GS115 ΔPp*spi1* MS1strains. The same control image was used as a representative image. Strains were incubated at 30°C for 48 h for observation. (**D**) SEM. (E) TEM images of the GS115 WT, GS115 ΔPp*spi1*, GS115 ΔPp*spi1* S, and GS115 ΔPp*spi1* MS1cells. The white arrows indicate irregular cell morphology and the red arrows indicate cell wall mannoprotein layer.

In *S. cerevisae*, the cell wall glycoprotein ScSpi1 was reported to be involved in weak acid resistance and was just induced during the stationary phase of growth (26). However, the expression level of Pp*SPI1* remains stable throughout all of the growth phases (Fig. S4). In order to gain insights into the role of PpSpi1 in cell wall integrity, a GS115 ΔPp*spi1* mutant was constructed. This mutant showed growth delay (Fig. 5A) and weaker resistance to cell wall/membrane perturbing agents (Fig. 5C). It also presented a rough cell surface, abnormal bud scars, irregular cell shape, a thinner, and sparser mannoprotein layer, similar to those of Glyco4 (Fig. 5D and E). The PpSpi1-complemented strain GS115ΔPp*spi1* S (Table S4, complementation of PpSpi1 in the GS115ΔPp*spi1* strain) exhibited not only a recovered growth rate similar to that of the GS115 WT strain (Fig. 5A) but also the increased resistance to SDS, caspofungin, congo red, or CFW (Fig. 5C). The morphological defects and thinning of the mannoprotein layer of cell wall were also ameliorated (Fig. 5D). Although the N-glycosylation sites mutated protein (PpSpi1^N21D, N51D^) is still localized to the outer layer of the cell wall (Fig. 5B) and has similar GFP fluorescence intensity (Fig. 5), the PpSpi1^N21D, N51D^ complemented strain GS115ΔPp*spi1* MS1 (Table S4, complementation of PpSpi1^(N21D, N51D)^ in the GS115ΔPp*spi1* strain) exhibited no obvious change in phenotypes compared to the GS115ΔPp*spi1* strain (Fig. 5). These results demonstrate that PpSpi1 is a major component of the mannoprotein layer and important for cell wall integrity.

### Overexpression of Pp*SPI1* gene in Glyco4 could partially ameliorate the growth delay and rescued the cell wall defects

**Fig 6:**
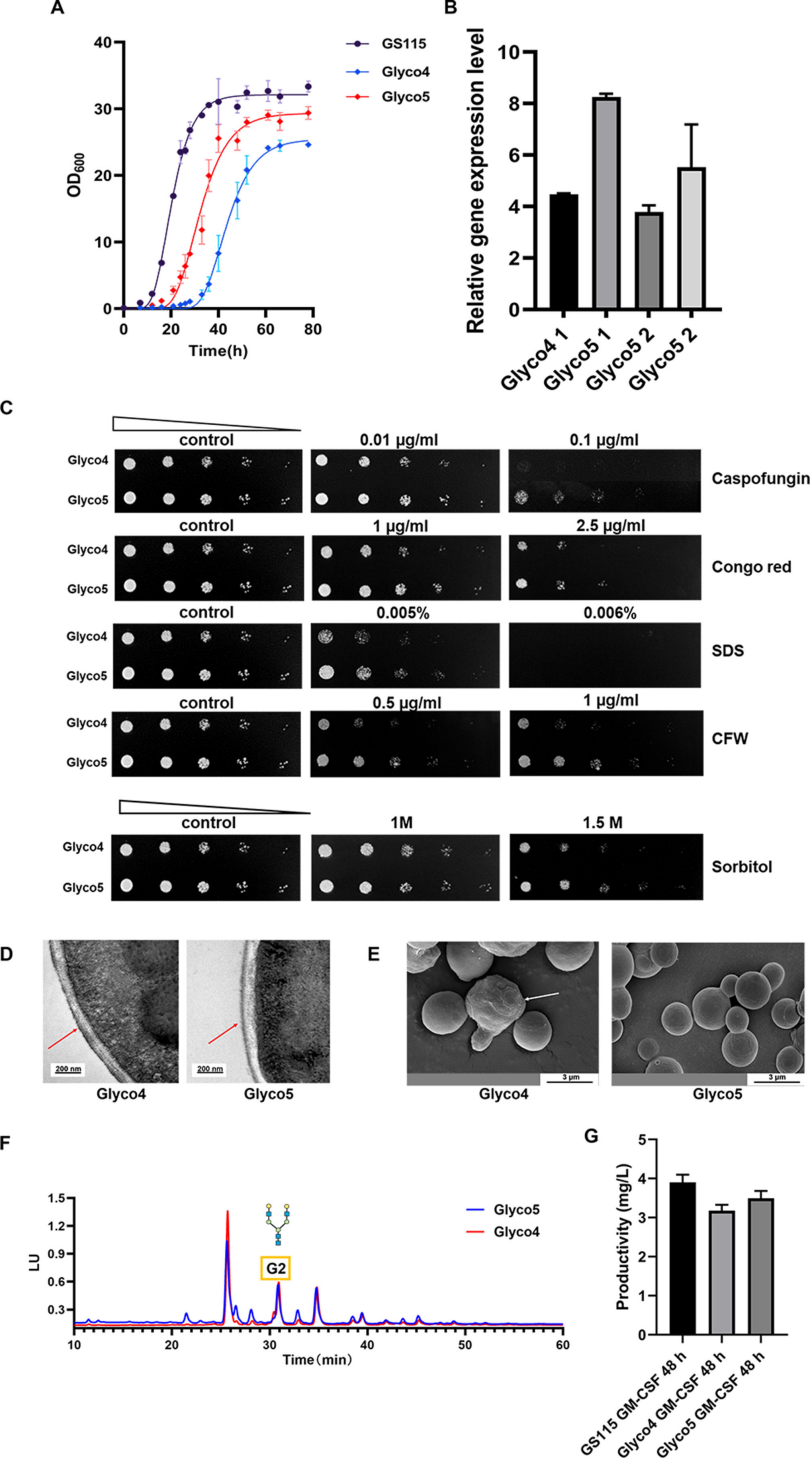
The phenotypes of Glyco5. (**A**) The growth curves of Glyco4 and Glyco5 strains. The GS115 and Glyco4 strains were cultivated in 50 mL YPD broth, at 30°C, 220 rpm. (**B**) Real-time quantitative PCR (RT-qPCR) analysis of the relative expression levels of Pp*SPI1* in Glyco4 and Glyco5, 1 and 2 represent the timepoint at the lag phase and log phase, respectively. (**C**) Spot plate analysis for caspofungin, Congo red, SDS, CFW, and sorbitol resistance of Glyco4 and Glyco5. The same control image was used as a representative image. The strains were incubated at 30°C for 72 h for observation. (**D**) TEM images of Glyco4 and Glyco5. The red arrows indicate the mannoprotein layer on cell wall surface. (**E**) SEM images of Glyco4 and Glyco5. The white arrow indicates the irregular cell morphology. The white arrow indicates the irregular cell morphology. (**F**) FLD-HPLC chromatogram of the N-glycans generated by Glyco4 and Glyco5 after methanol induction for 48 h under bioreactor cultivation. (**G**) GM-CSF productivity of the GS115 WT, Glyco4, or Glyco5 strains cultured in bioreactor.

As it mentioned above, the expression level of Pp*SPI1* was down-regulated in Glyco4, resulting in cell wall defects and growth delay. In order to alleviate the drawbacks, an additional Pp*SPI1* gene copy was integrated into Glyco4 to generate a Glyco5 strain. The growth delay of Glyco5 was ameliorated (Fig. 6A), and the expression level of Pp*SPI1* was twice as high as in Glyco4 and close to the level in the GS115 WT strain (Fig. 6B). Glyco5 showed improved resistance to caspofungin, congo red, SDS, CFW, and osmotic stress (Fig. 6C). According to the images from TEM and SEM, the mannoprotein layer of Glyco5 becomes thicker, more uniform (Fig. 6D), and the cell is smoother and rounder (Fig. 6E). In addition, the N-glycan structures of glycoprotein produced in Glyco5 were similar to those of Glyco4 (Fig. 6F). As shown in Fig. 6G, under bioreactor culture condition, the productivity of GM-CSF in Glyco5 was increased compared with that in Glyco4. It indicates that overexpressing Pp*SPI1* in Glyco4 could not only ameliorate the growth delay and rescue the cell wall defects but also increase protein productivity.

### A conserved sequence fragment in PpSpi1 is widespread in fungi

**Fig 7:**
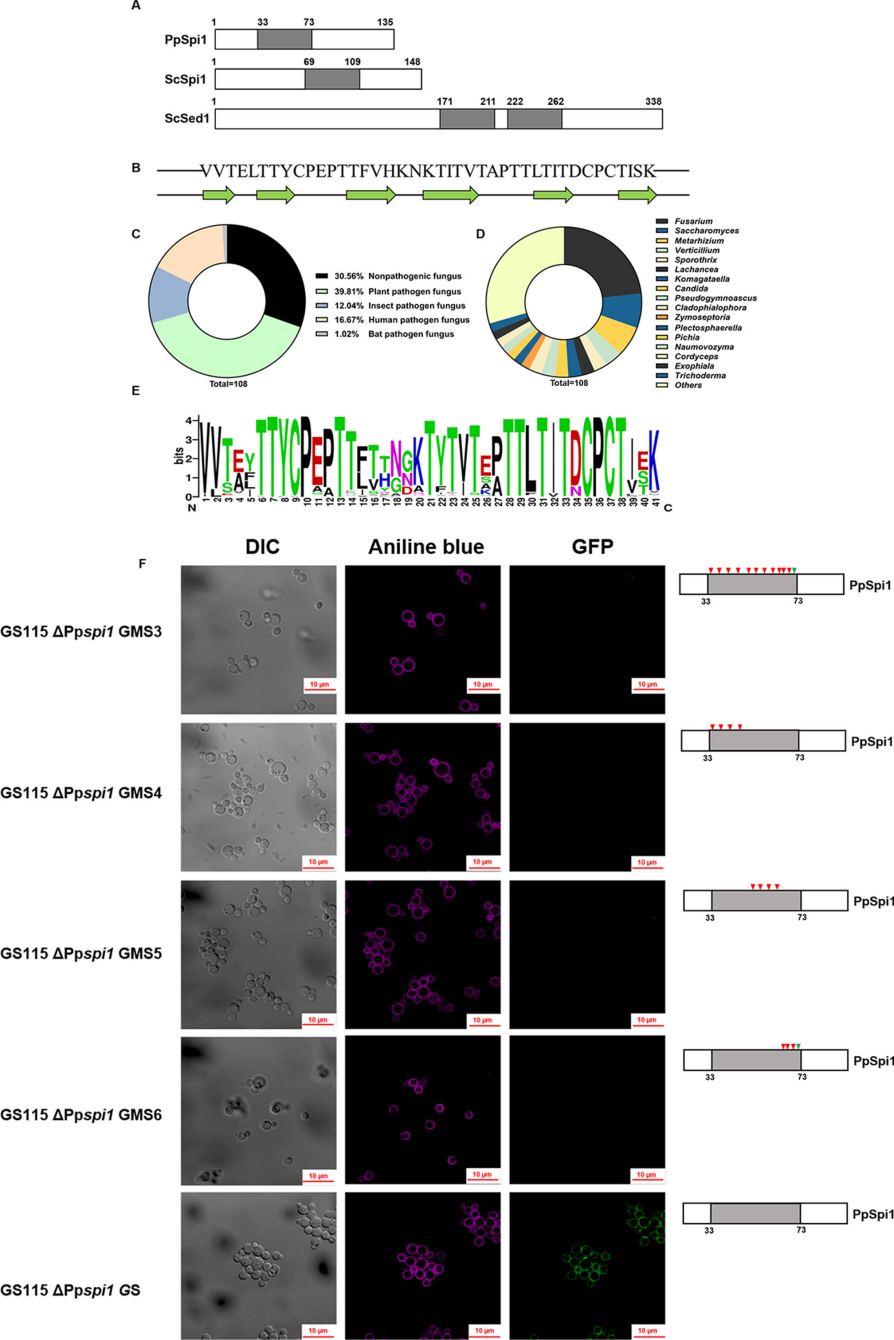
Feature and distribution of the conserved fragment of 41-residue. (**A**) Schematic of the conserved fragment distribution in PpSpi1, ScSpi1, and ScSed1; the gray rectangle represents the 41-residue fragment. (**B**) Secondary structure prediction of the conserved fragment in PpSpi1 by jpred4 (29). (**C**) Alignment of the conserved fragment containing proteins from 108 fungal species; the sequence logo was created using WebLogo. (**D**) The percentage of the fungal species with protein containing the conserved fragment. (**E**) The genus distribution of the fungi with protein containing the conserved fragment. (**F**) Fluorescence detection of various GFP-PpSpi1 mutant complemented strains. The gray rectangle represents the 41-residue fragment, and the triangles represent 12 conserved threonine sites in order (38, 39, 45, 46, 53, 55, 57, 60, 61, 63, 65, 70). The red triangle indicates a T to N mutation, and the green triangle indicates a T to Q mutation. EGFP: Ex/EM 488 nm/509 nm, detection wavelength 450–700 nm, AF405: Ex/EM 401 nm/422 nm, detection wavelength 400–496 nm. The blue fluorescence of aniline blue was converted to purple by using ZEN2 software. Adobe Photoshop (PS) CC 2018 was used for image editing.

Interestingly, the sequence alignment identified a conserved threonine-rich fragment of 41-amino acid residue which occurs once in PpSpi1 and ScSpi1 and twice in ScSed1 (Fig. 7A). ScSed1 is a paralog of ScSpi1 that arose through the whole-genome duplication (WGD) (27). Like ScSpi1, ScSed1 was also confirmed to be a GPI-anchored cell wall glycoprotein of *S. cerevisiae* and involved in lytic enzyme resistance (28). This fragment was predicted to contain six β-sheets (Fig. 7B). Using the conserved fragment alignment, 202 fungal proteins distributed in 108 species and 49 genera were identified from the NCBI, FungiDB, and CGD databases (Table S3). Each of these proteins was predicted to contain one GPI site, one secretion signal peptide, and most of them have at least one predicted N-glycosylation site, implying these proteins are potential cell wall-anchored proteins. Some predicted N-glycosylation sites happen to be located within the conserved fragments, such as the second N-glycosylation site in PpSpi1. All of 108 species belong to the Phylum *Ascomycota;* 69.4% of them were reported to be the pathogenic fungi (Fig. 7C), including plant pathogens such as *Fusarium* and *Verticillium* species, human pathogens such as *Candida albicans* and *Candida glabrata*, and entomopathogenic fungi such as *Metarhizium* species (Table S3; Fig. 7D). Among the strains belonging to the 49 genera, the proportion of *Fusarium* strains is the highest (Fig. 7D). To evaluate the feature of fragments, a sequence logo for 202 conserved fragments was generated (Fig. 7E), in which most of the threonine residues were highly conserved (the 6th, 7th, 13th, 14th, 21th, 23th, 25th, 28th, 29th, 31th, 33th, and 38th threonine residues in the fragment of 41-residue).

In order to investigate the importance of highly conserved threonine residues in the fragment, four mutation strategies for PpSpi1 were performed: (i) Among the 12 conserved threonine residues in this fragment, the 38th threonine residue was replaced by glutamine (Q) to avoid the formation of new N-glycosylation site, and the other eleven threonine residues were replaced by asparagine (N). (ii) Only the 6th to 14th threonine residues were substituted by N. (iii) Only the 21th to 28th threonine residues were substituted by N. (iv) Only the 29th to 33th threonine residues were substituted by N, and the 38th threonine residue was substituted by Q. The four PpSpi1 variants were, respectively, complemented into the GS115 ΔPp*spi1* strain to generate the GS115 ΔPp*spi1* MS3\MS4\MS5\MS6 mutants (Table S4). As shown in Fig. S6, the growth curves of these mutants were similar to that of the GS115 ΔPp*spi1* strain but slower than that of the GS115 WT strain. In addition, GFP fusion expression of four PpSpi1 variants is very likely lead to misfolding of PpSpi1 (Fig. 7F). This further illustrates the importance of conserved fragment in the structure and function of PpSpi1. Because the overwhelming majority of above 202 fungal proteins are uncharacterized, the functional and structural role of this conserved fragment is still uncertain. Wide distribution of this conserved fragment among fungal proteins suggests an important function and would be worth a future in-depth study.

## DISCUSSION

Production of therapeutic glycoproteins in yeasts has a natural disadvantage because of their inability to modify proteins with human-like N-glycan structures. In recent years, the humanization of N-glycosylation pathways in yeast and other fungi species has shown significant promise. However, the N-glycosylation engineered yeast strains lacking characteristic hyper-mannosylation reactions always exhibit morphological defects and poor growth (17, 30, 31).

In this study, a *P. pastoris* mutant Glyco4, which had undergone humanization of N-glycosylation pathway and could successfully generate human-like N-glycoprotein, was carefully evaluated. Comparative transcriptomics reveals that N-glycosylation engineering could affect the expression behaviors of numerous biological pathways in *P. pastoris*, especially the glycolysis pathway, and could induce the CWI response. This is consistent with the observation that Glyco4 displays a remarkable growth delay and cell wall defects. PpSpi1, a GPI-anchored cell wall glycoprotein with very high expression level, was confirmed to play a critical role in maintaining cell wall stability by helping to form the dense mannoprotein layer on the cell wall surface. The down-regulation of Pp*SPI1* after N-glycosylation engineering was proven to be the main cause of cell wall defects in Glyco4. Moreover, overexpressing Pp*SPI1* in Glyco4 could partially rescue the cell wall defects and improve the growth of *P. pastoris*, also improving its resistance to cell wall perturbing agents and osmotic stress. Thus, overexpressing PpSpi1 as a repairing protein would significantly facilitate industrial applications of *P. pastoris* in manufacture of therapeutic glycoproteins.

*P. pastoris* Spi1 (PpSpi1) has about 49.1% sequence identity to *S. cerevisiae* Spi1 (ScSpi1). A conserved sequence of 41-residue was identified in both ScSpi1 and PpSpi1. And, it was also found to occur twice in ScSed1. This conserved sequence was not found in any other protein in *P. pastoris,* most likely because *P. pastoris* is a non-WGD (32) species and has no paralog of PpSpi1 identified in its genome. According to the previous reports, ScSpi1 was strongly induced in the stationary phase (33) and played a prominent role in the development of *S. cerevisiae* resistance to lipophilic weak-acid food preservatives (34). And, ScSed1 was also confirmed to be a major cell wall protein of *S. cerevisiae* during the stationary phase (28). This suggests an additive effect conferred by ScSed1 and ScSpi1 in *S. cerevisiae*. Unlike Sc*SPI1*, the transcriptional level of Pp*SPI1* is stable in all growth phases of *P. pastoris* (Fig. S4). The fact that there is no paralog of PpSpi1 in *P. pastoris* suggests that PpSpi1 may play a more important role than ScSpi1 in cell wall protection. As the regulatory mechanism of Pp*SPI1* is still unclear, the reason why Pp*SPI1* is down-regulated in the Glyco4 mutant is very attractive and will be the focus of our future attention.

Interestingly, the proteins containing a conserved sequence of 41-residue were found to be widespread in *Ascomycota,* including *Candida* sp., *Fusarium* sp., and *Claviceps purpurea* (Table. S3). As mentioned above, this conserved sequence is threonine rich, implying these threonine residues might be the potential O-linked glycosylation sites (35). As O-glycosylation of cell wall proteins is important for many biological processes, we believe that the homologs of PpSpi1 in other fungi might also be involved in crucial processes like cell wall integrity.

Although the humanization of N-glycosylation pathway in yeast has gone through many years, there are still many unsolved problems. Our work ultimately improves the properties of glycoengineering *P. pastoris* by characterization and overexpression of an important cell wall glycoprotein, which is an attempt and more related research is needed to advance this field in the future.

## MATERIALS AND METHODS

### Strains, vectors, and media

The strategy to construct Glyco4 strain followed previous reports (15, 36) with minor modification. Briefly, the SuperMan5 HIS− strain which generates a more uniform Man5 glycosylation at N-linked glycosylation site was obtained from Pichia GlycoSwitch (https://pichia.com/try-pichia/try-pichia-glycoswitch/). In this study, three endogenous genes, *BMT2* (*PAS_chr4_0450*), *MNN4-3* (*PAS_chr2-1_0706*)*,* and *PNO1* (*PAS_chr1-4_0410*)*,* were knocked out by CRISPR-Cas9 (37, 38). The sgRNA sequences and the donator vectors for repairing are listed in Table S4. The genes encoding Yea4 (*Kluyveromyces lactis*, UDP-N-acetylglucosamine transporter), Gnt1 (*Homo sapiens,* β-1,2-N-acetylglucosaminyltransferase I), Gnt2 (*Rattus norvegicus*, β-1,2-N-acetylglucosaminyltransferase II), Mns2 (*Drosophila melanogaster,* α-1,3/6-Mannosidase), Uge1 (*Schizosaccharomyces pombe*, UDP-glucose 4-epimerase), UgalT (*D. melanogaster*, UDP-galactose transporter), and Galt1 (*H. sapiens*, β-1,4-galactosyltransferase) were introduced into the genome of SuperMan5 HIS^−^ strain by CRISPR-Cas9 to generate the Glyco4 strain. The related locus and sgRNA sequences were described in the donor vectors listed in Table S4.

For construction of the GS115ΔPp*spi1* mutant, Pp*SPI1* targeting sgRNA-Cas9 vector pΔPp*spi1* sgRNA Cas9 and donor DNA cassettes pGGA-ΔPp*spi1* HR-BleR (Table S4) were co-transferred into the GS115 WT strain and screened by colony PCR.

For construction of the various Pp*SPI1* complemented strains, including GS115 ΔPp*spi1* S/MS1/MS3/MS4/MS5/MS6/GS/GMS1/GMS2/GMS3/GMS4/GMS5/GMS6 (Table S4), the *P. pastoris* neutral site IV-9 (38) targeting sgRNA-Cas9 vector IV-9 sgRNA Cas9 and the donor vectors (Table S4) were transferred into the GS115 ΔPp*spi1* strain and screened by colony PCR. So, did the Glyco5 strain construction process. Based on a similar method, Pp*SPI1* gene was introduced into Glyco4 to construct the Glyco5 strain.

The GS115 WT and its derivate strains were cultivated in yeast extract-peptone-dextrose (YPD) medium at 30°C. Yeast cells were pre-cultivated in buffered glycerol-complex medium (BMGY) at 30°C, 220 rpm. Buffered methanol-complex medium (BMMY), synthetic-complete (SC) medium, and Luria-Bertani medium were used for protein expression, spot plate analysis, and *E. coli* (TG1) culturing, respectively.

### Measurement of yeast growth

Yeast strains were pre-cultured in 5 mL YPD broth at 30°C, 220 rpm until stationary phase. Subsequently, the inoculated strains were transferred to an initial OD_600_ of 0.1 in 50 mL YPD fresh broth, cultivated at 30°C, 220 rpm. The optical absorbance (OD_600_) of the culture was measured by EVOLUTION 220 spectrophotometer (Thermo Fisher Scientific Inc, USA) every 2 h. Each strain had three replicates.

### Flocculation ability detection

The GS115 WT and Glyco4 strains were pre-cultured in 5 mL YPD broth at 30°C, 220 rpm to stationary phase. Subsequently, the inoculated GS115 WT and Glyco4 cells were transferred to 50 mL YPD fresh broth at an initial OD_600_ of 0.1, cultivated at 30°C, 220 rpm for 30 or 70 h, respectively (the timepoint that the GS115 WT or Glyco4 cells enter into their stationary phase). Shaking the conical flask after cultivation to ensure the fermentation broth is well mixed and then keeping it at room temperature for 10 min.

### RNA-seq analysis and KEGG pathway enrichment

The total RNA of GS115 WT and Glyco4 strains were prepared from cells cultured in 50 mL YPD broth at 30°C, 220 rpm, and two replicates of yeast cells (total OD_600_ = 16) were harvested at three sampling timepoints (respectively, represents the lag phase, log phase, or stationary phase of yeast growth). Total RNA isolation was performed according to a previous report (39). The BGISEQ-500 platform (BGI Shenzhen, China) was used for RNA-seq. After that, RNA-seq analysis was performed, and the low-quality reads and reads containing adapters were removed to get the clean reads data. Then, aligned paired-end clean reads to the available reference genome of *Komagaetella phaffii* using HISAT (40). RSEM was employed to quantify the gene expression level in FPKM (number of fragments per kilobase of the transcript sequence per million base pairs sequenced) (41). The differential expression analysis was performed using the DESeq2 (42). Genes with log_2_ fold change (Glyco4 relative to GS115 WT) (log_2_FC) ≥1 (upregulated) or log_2_FC ≤1 (down-regulated), *Q*-value (adjusted *P*-value by FDR) (43) ≤0.05 were considered differentially expressed genes in comparative analysis. The *R*’s phyper function was used for KEGG pathway enrichment analysis of down and up-regulated genes. *P*-value was calculated by the previously reported method (42).

### Real-time quantitative PCR analysis

The real-time quantitative PCR (RT-qPCR) analysis was performed according to the previous report (39). The *ACT1* gene was chosen as an internal control to normalize the relative expression levels of target genes. All reactions were performed in three replicates.

### Susceptibility tests to cell wall perturbing agents

Yeast strains were cultivated in 50 mL YPD broth to the logarithmic phase. The yeast cells (OD_600_ = 1) were harvested by centrifugation at 12,000 rpm for 30 s and resuspended with 10 mL sterile distilled water. Serial fivefold dilutions were performed, and 3 µL of each diluent was spotted onto the SC plates containing congo red, caspofungin, or SDS. The above plates were incubated at 30°C.

### N-glycan analysis of the recombinant glycoprotein produced by *P. pastoris*

The N-glycan analysis process was performed according to a previous report (44) with several modifications. Briefly, 50 µg of glycoprotein GM-CSF (granulocyte-macrophage colony-stimulating factor) produced by the GS115 WT, Glyco4, or Glyco5 strains was treated with PNGase F for overnight. The reaction mixture was then directly labeled with 2-aminobenzamide for 4 h at 65°C. The labeled N-glycans were isolated by the solid-phase extraction kit and, subsequently, analyzed by HPLC–FL–MS.

### TEM and SEM analysis

The strains were cultivated in 50 mL YPD broth at 30°C, 220 rpm. Yeast cells (total OD_600_ = 20) at logarithmic phase were harvested by centrifugation. Then, TEM and SEM were performed according to the previous reports (45, 46).

### Yeast cell wall staining and optical microscopy analysis

Aniline blue staining of the cell wall β-1,3-glucan was carried out according to a previous report (47). The confocal images were acquired on a Zeiss LSM 800 confocal microscope with a 100 × oil immersion objective; EGFP: Ex/EM 488 nm/509 nm, detection wavelength 450–700 nm, AF405: Ex/EM 401 nm/422 nm, detection wavelength 400–496 nm. Images were analyzed using ZEN2 software.

### Cell wall isolation

Yeast cell wall isolation was performed as described previously (48). Yeast cells cultivated in 50 mL YPD broth were harvested (total OD_600_ = 150) at exponential phase by centrifugation and resuspended with 5 mL cold distilled water. The cell pellets were centrifuged again and resuspended with 750 µL cold Tris-HCl buffer (10 mM, pH 8.0) containing 1 g acid-washed glass beads. The yeast cells were broken using a mechanical bead beater setting 50 HZ, for six 60 s periods alternating with 60 s intervals on ice. The cell fragments were washed with 750 µL Tris-HCl buffer (10 mM, pH 8.0) and centrifuged. The supernatant was then transferred into a 15-mL falcon tube and centrifuged at 9,000 rpm for 15 min. The pellets were resuspended by 400 µL Tris-HCl buffer and freeze-dried overnight. The resulted cell wall fragments were stored at −20°C.

### Acidic hydrolysis and analysis of the reducing sugars

Acid hydrolysis of the cell wall polysaccharides was performed as described previously (48). After that, the hydrolysate was cooled down and added into three volumes of acetonitrile. The mixture was centrifuged (12,000 rpm, 10 min), injecting 2 µL supernatant for LC-MS detection. The mannose, glucose, and N-GlcNAc standard solution (50 µg/mL, 50 µg/mL, 25 µg/mL, respectively, which also contained the fucose internal standard) were detected too.

LC/MS/MS analysis was performed using an Agilent 6,460 triple quadrupole mass spectrometer (Agilent Technologies, USA) equipped with an electrospray ionization (ESI) source and operated in the negative single-ion monitoring (SIM) mode. Agilent Mass Hunter Workstation was used for data acquisition and processing. Nitrogen was used as the sheath gas and drying gas. The nebulizer pressure was set to 45 psi, and the flow rate of drying gas was 5 L/min. The flow rate and temperature of the sheath gas were 11 L/min and 350°C, respectively. Chromatographic separation was carried out on an Agilent HILIC column (100 × 2.1 mm, 3.5 µm). The HPLC mobile phases consisted of acetonitrile and 0.2% aqueous ammonia solution (pH 10), for gradient elution. The flow rate was set at 0.13 mL/min. Mass spectrometric detection was completed by use of a ESI source in negative SIM mode.

### Bioinformatics analysis of conserved sequence

The 41-residue fragment of PpSpi1 was blasted in NCBI, FungiDB, and CGD. The resulted protein sequences which contained the conserved fragment with over 50% identity to the 41-residue fragment of PpSpi1 were downloaded and listed in Table S3. The signal peptide of each protein was predicted by SignalP 6.0 (http://www.cbs.dtu.dk/services/SignalP/). The GPI modification sites and the N-glycosylation sites were predicted on the web server (http://mendel.imp.ac.at/gpi/fungi/gpi_fungi.html) and by NetNGlyc 1.0 (https://services.healthtech.dtu.dk/service.php?NetNGlyc-1.0), respectively. The proteins without predicted signal peptide and GPI modification sites were eliminated. All of the conserved fragments were aligned by ClustalW (49) to create a sequence LOGO by using WebLogo (50).

### Bioreactor cultivation

Bioreactor cultivation was performed in a 3-L bioreactor (T&J-IntelliFermB, china), and the working volume was 1.5 L. NH_4_OH was used as nitrogen source and to set and maintain pH 6, 30°C. A minimum DO of 25% was set under cascade control setting. Preculture was grown in 150 mL BMGY broth until OD_600_ = 10 at 30°C, 220 rpm and then aseptically transferred to 1.5 L BMMY media for batch cultivation and grown until the glycerol got depleted. The batch mode was carried out for biomass production until the available glycerol got depleted as indicated by a DO spike. Thereafter, 1 mL/h of methanol was fed for 4 h to let the culture adapt the methanol and then set the feed rate to 3 mL/h. Keep this mode throughout the remainder of the fermentation. Above conditions and processes were used for GM-CSF production.

### Purification of GM-CSF protein

Fifty milliliters of fermentation broth were harvested and transferred into a centrifuge tube, centrifuged at 4°C, 10,000 rpm for 15 min. The supernatant was transferred to a 250-mL beaker containing 26.31 g ammonium sulfate and gently stirred until it completely dissolved. After leaving at 4°C overnight, the mixture was transferred to the centrifuge tubes and centrifuged at 4℃, 10,000 rpm for 15 min. The pellet was resuspended with 20 mM pH 7.6 Tris-HCl buffer and then desalinized by the Ultra Centrifugal Filter Units (Amicon Ultra-15, Millipore). The resuspend supernatant was transferred to another Ultra Centrifugal Filter Units and centrifuged at 4°C, 10,000 rpm for 15 min. After the effluent was discarded, 15 mL of 20 mM Tris-HCl buffer containing 10 mM imidazole was added and then centrifuged at 10,000 rpm, 4°C for 15 min. Repeat this step three times. Finally, the concentrated liquid was recycled for Ni-NTA affinity chromatography.

The Ni-NTA affinity chromatography was performed according to a previous report (51). The protein concentration was tested by modified Bradford Assay Kit (C503041, Sangon Biotech).

### Flow cytometric analysis

The yeast strains were cultivated in 50 mL YPD broth until OD_600_ = 5 at 30°C, 220 rpm. A total of 3 × 10^7^ cells were harvested by centrifugation at 5,000 rpm, 4°C for 5 min. The pellets were washed by 500 µL cold 1 × PBS buffer twice and fixed by paraformaldehyde for 30 min. The samples were washed with 1 × PBS buffer again and 3 × 10^6^ cells were harvested for analysis. CytoFLEX LX flow cytometer (Beckman Coulter) was used for cellular GFP fluorescence determination, and the process was according to a previous report (52).

## Data Availability

The sequence reads of GS115 WT and Glyco4 obtained by RNA-seq were deposited in the SRA database under accession number PRJNA890185.

## SUPPLEMENTAL MATERIAL

Fig. S1 mbio.00617-23-s0001.pdf Mass spectra of G2 type N-glycan derived from GM-CSF produced by the Glyco4 strain.

Fig. S2 mbio.00617-23-s0002.pdf The flocculaion ablity and transcriptional profile of the GS115 WT and Glyco4 strains.

Fig. S3 mbio.00617-23-s0003.pdf Sequence alignment of PpSpi1 and ScSpi1.

Fig. S4 mbio.00617-23-s0004.pdf The relative expression levels of PpSPI1 in the GS115 WT strain at different sampling timepoints.

Fig. S5 mbio.00617-23-s0005.pdf GFP fluorescence of the GS115 ΔPp*spi1*, GS115 ΔPp*spi1* GS, or GS115 ΔPp*spi1* GMS1 strains.

Fig. S6 mbio.00617-23-s0006.pdf Comparison of growth among the PpSpi1 mutants complemented strains, GS115 ΔPp*spi1* and GS115 WT strains.

Table S1 mbio.00617-23-s0007.xlsx The differentially expressed genes in Glyco4 compared with the GS115 WT strain at three sampling timepoints.

Table S2 mbio.00617-23-s0008.xlsx The expression levels of predicted GPI proteins.

Table S3 mbio.00617-23-s0009.xlsx Statistics of genus and species having the proteins with conserved 41-residue fragment.

Table S4 mbio.00617-23-s0010.pdf Strains and vectors used in this study.

ASM does not own the copyrights to Supplemental Material that may be linked to, or accessed through, an article. The authors have granted ASM a non-exclusive, world-wide license to publish the Supplemental Material files. Please contact the corresponding author directly for reuse.
