# Supplementary material for: Impact of PpSpi1, a glycosylphosphatidylinositol-anchored cell wall glycoprotein, on cell wall defects of N-glycosylation-engineered Pichia pastoris
Source: mBio. 2023 Aug 22;14(5):e00617-23. doi: 10.1128/mbio.00617-23 (PMC10653784; doi:10.1128/mbio.00617-23)
Supplement: Fig. S1 — Mass spectra of G2 type N-glycan derived from GM-CSF produced by the Glyco4 strain. [file mbio.00617-23-s0001.pdf]

A

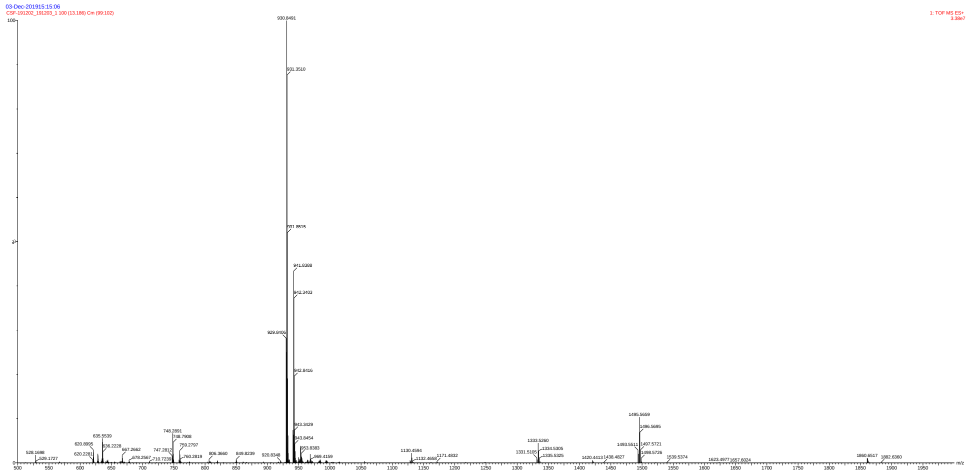

B

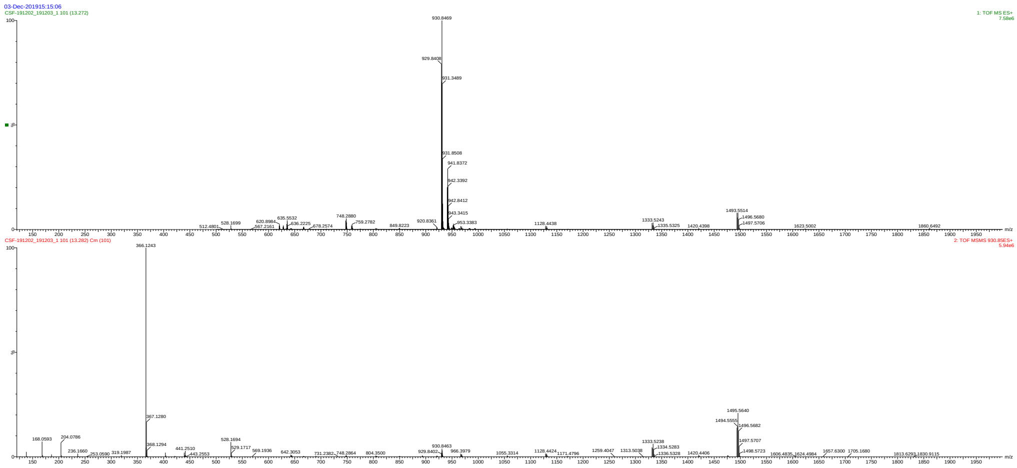

**Fig. S1** Mass spectra of G2 type N-glycan derived from GM-CSF produced by the Glyco4 strain. A and B represent MS and MS-MS respectively,The target Mass-to-core ratio ($[M+2H]^{2+}$) of G2 is 930.89, and the detected Mass-to-core ratio of G2 is 930.85, (1657.63 ($H_5N_3$-PROC) 1495.56 $[748.29]^{2+}$ ($H_4N_3$-PROC) 1333.52 $[667.26]^{2+}$ ($H_3N_3$-PROC) 1171.48 ($H_2N_3$-PROC) 1130.46 ($H_3N_2$-PROC) 968.41 ($H_2N_2$-PROC) 806.37 ($H_1N_2$-PROC) 644.32 ($N_2$-PROC) 441.25 ($N_1$-PROC) 1420.44 ($H_5N_3$) 1217.38 ($H_5N_2$) 1055.33 ($H_4N_2$) 893.28 ($H_3N_2$) 690.21 ($H_3N_1$) 528.17 ($H_2N_1$) 366.12 ($H_1N_1$)), H= Hexose, N= N-Acetyl hexosamine)
