# Supplementary material for: Impact of PpSpi1, a glycosylphosphatidylinositol-anchored cell wall glycoprotein, on cell wall defects of N-glycosylation-engineered Pichia pastoris
Source: mBio. 2023 Aug 22;14(5):e00617-23. doi: 10.1128/mbio.00617-23 (PMC10653784; doi:10.1128/mbio.00617-23)
Supplement: Fig. S2 — The flocculaion ablity and transcriptional profile of the GS115 WT and Glyco4 strains. [file mbio.00617-23-s0002.pdf]

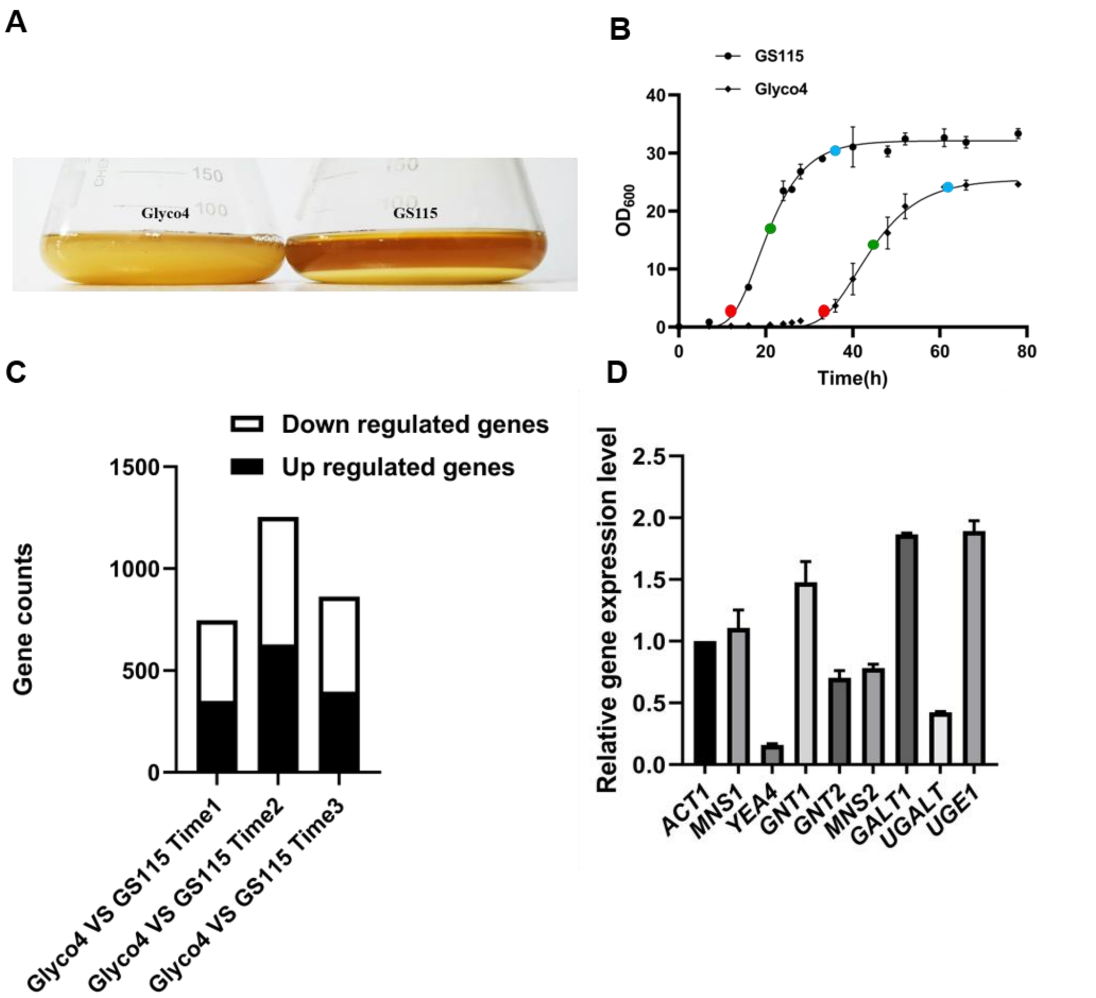

Fig. S2 The flocculaion ablity and transcriptional profile of the GS115 WT and Glyco4 strains. **A.**The flocculation ability of GS115 WT and Glyco4 strains. The Glyco4 and GS115 strains were cultivated in 50 ml YPD broth, at 30°C, 220 rpm. Details of the experiment are presented in the 'method and material' section. **B.** Three sampling timepoints for RNA-seq (The red, green or blue solid circles respectively represent the timepoint at the lag phase, log phase or stationary phase of yeast growth). The GS115 and Glyco4 strains were cultivated in 50 ml YPD broth, at 30°C, 220 rpm. The initial inoculation concentration was OD_{600}=0.1. **C.** Histogram of the differentially expressed genes. The differential expression analysis was performed by using the DESeq2. Genes with Log_2 fold change (Glyco4 relative to GS115, Log_2FC) ≥1 (upregulated) or Log_2FC ≤1 (down-regulated), Q-value (Adjusted P-value by FDR ≤ 0.05. **D.** The relative expression levels of eight exogenous N-glycosylation-related genes in Glyco4 which cultivated in 50 ml YPD broth, sampling at logarithmic phase, and exogenous gene expression relative to *ACT1* was normalized by $2^{\wedge-(Ctgene-Ct\,ACT1)}$.
