# Supplementary material for: Impact of PpSpi1, a glycosylphosphatidylinositol-anchored cell wall glycoprotein, on cell wall defects of N-glycosylation-engineered Pichia pastoris
Source: mBio. 2023 Aug 22;14(5):e00617-23. doi: 10.1128/mbio.00617-23 (PMC10653784; doi:10.1128/mbio.00617-23)
Supplement: Fig. S3 — Sequence alignment of PpSpi1 and ScSpi1. [file mbio.00617-23-s0003.pdf]

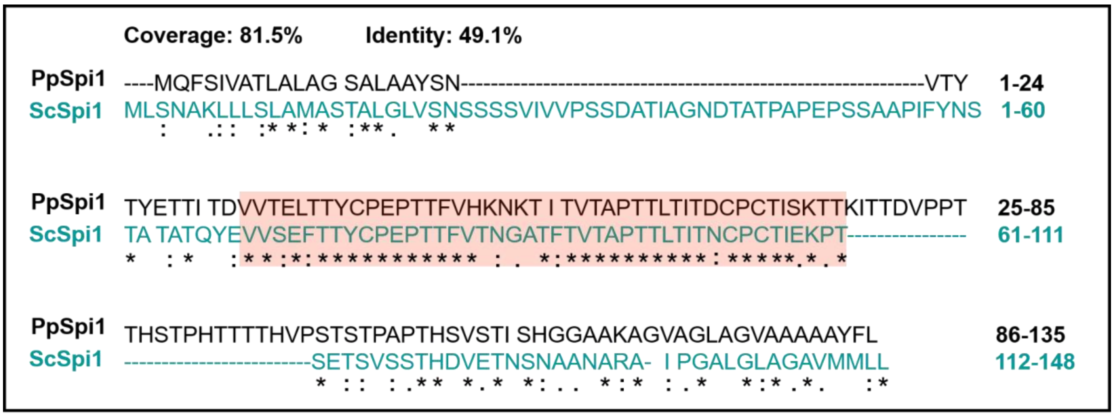

Fig. S3 Sequence alignment of PpSpi1 and ScSpi1. The brick-red block represents the conserved sequence fragment (41-residues) in PpSpi1 and ScSpi1
