# Supplementary material for: Impact of PpSpi1, a glycosylphosphatidylinositol-anchored cell wall glycoprotein, on cell wall defects of N-glycosylation-engineered Pichia pastoris
Source: mBio. 2023 Aug 22;14(5):e00617-23. doi: 10.1128/mbio.00617-23 (PMC10653784; doi:10.1128/mbio.00617-23)
Supplement: Fig. S4 — The relative expression levels of PpSPI1 in the GS115 WT strain at different sampling timepoints. [file mbio.00617-23-s0004.pdf]

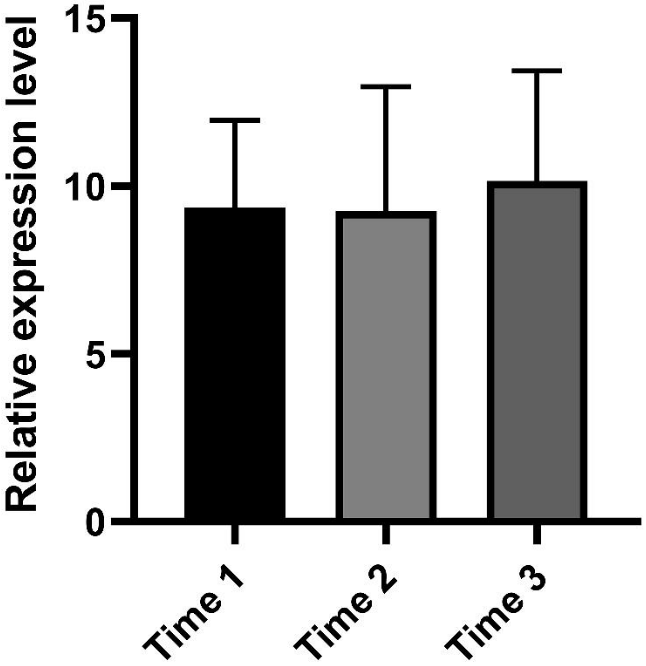

Fig. S4 The relative expression levels of Pp*SPI1* in the GS115 WT strain at different sampling timepoints. Time 1, 2, and 3 represent the samples harvested at OD_{600} = 3, 8, and 20, respectively. Error bars indicate the SD for samples tested in triplicate. The *ACT1* transcriptional level was detected and used as the internal control.
