# Supplementary material for: Impact of PpSpi1, a glycosylphosphatidylinositol-anchored cell wall glycoprotein, on cell wall defects of N-glycosylation-engineered Pichia pastoris
Source: mBio. 2023 Aug 22;14(5):e00617-23. doi: 10.1128/mbio.00617-23 (PMC10653784; doi:10.1128/mbio.00617-23)
Supplement: Fig. S5 — GFP fluorescence of the GS115 ΔPpspi1, GS115 ΔPpspi1 GS, or GS115 ΔPpspi1 GMS1 strains. [file mbio.00617-23-s0005.pdf]

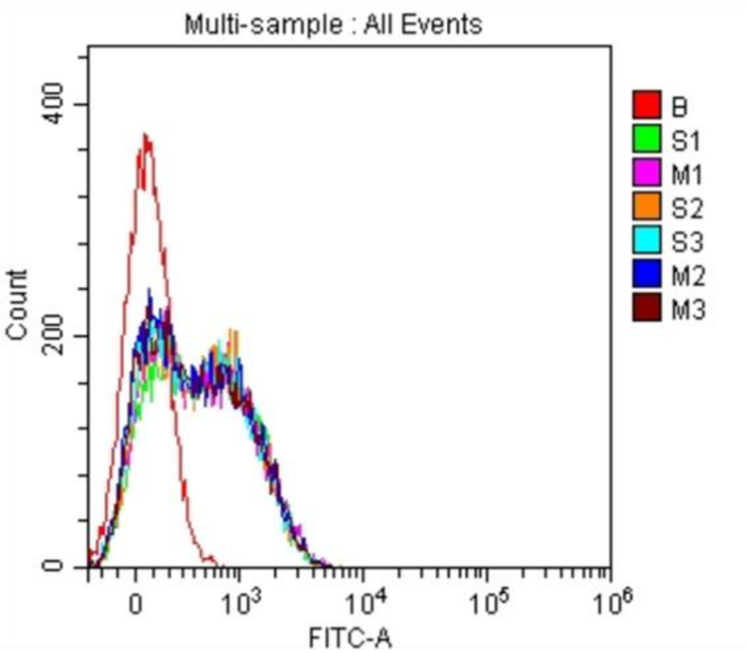

Fig. S5 GFP fluorescence of the GS115 ΔPp*spi1*, GS115 ΔPp*spi1* GS or GS115 ΔPp*spi1* GMS1 strains. The S1-S3 curves represent the GS115 ΔPp*spi1* GS strains for parallel respectively, and the M1-M3 curves represent the GS115 ΔPp*spi1* GMS1strains for parallel respectively. The B curve represents the GS115 ΔPp*spi1* strain as control.
