# Supplementary material for: Impact of PpSpi1, a glycosylphosphatidylinositol-anchored cell wall glycoprotein, on cell wall defects of N-glycosylation-engineered Pichia pastoris
Source: mBio. 2023 Aug 22;14(5):e00617-23. doi: 10.1128/mbio.00617-23 (PMC10653784; doi:10.1128/mbio.00617-23)
Supplement: Fig. S6 — Comparison of growth among the PpSpi1 mutants complemented strains, GS115 ΔPpspi1 and GS115 WT strains. [file mbio.00617-23-s0006.pdf]

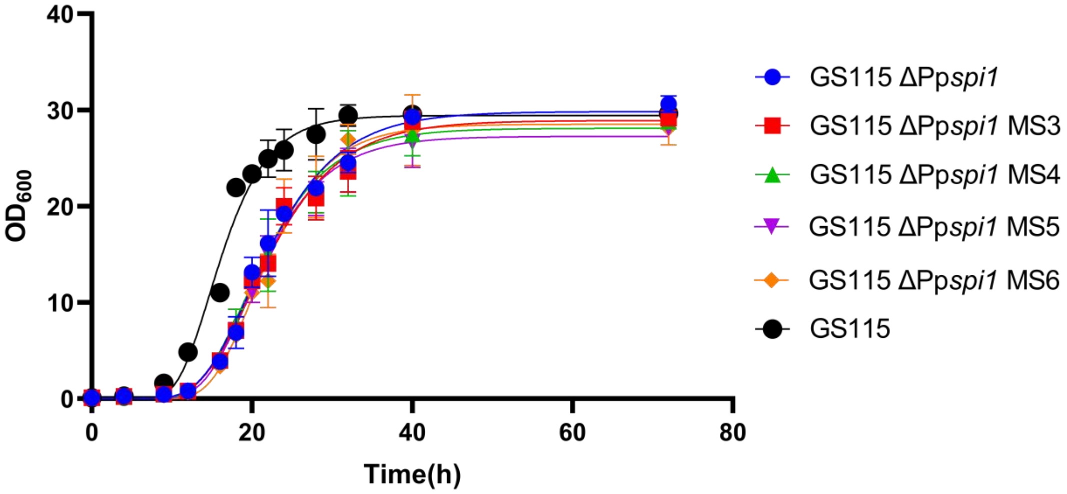

Fig. S6 Comparison of growth among the PpSpi1 mutants complemented strains, GS115 ΔPp*spi1* and GS115 WT strains. The strains were cultivated in 50 ml YPD broth, at 30°C, 220 rpm. All of the initial OD_{600} were 0.1.
