# Supplementary material for: Impact of PpSpi1, a glycosylphosphatidylinositol-anchored cell wall glycoprotein, on cell wall defects of N-glycosylation-engineered Pichia pastoris
Source: mBio. 2023 Aug 22;14(5):e00617-23. doi: 10.1128/mbio.00617-23 (PMC10653784; doi:10.1128/mbio.00617-23)
Supplement: Table S4 — Strains and vectors used in this study. [file mbio.00617-23-s0010.pdf]

Table S4 Strains and vectors used in this study

	Description	References
**Strains**		
E. coli TG1	Host strain for DNA clone	Novagen
P. pastoris GS115	his4-, the basic strain for this study	Invitrogen
SuperMan5	HIS^-, Och1-disruption with a pGAP-mannosidase (MnsI, *Trichoderma reesei*) expression cassette, blasticidin resistant	Pichia GlycoSwitch®
P. pastoris Glyco4	N-glycosylation engineering strain which can generate G2 type Glycan	This study
GS115 CSF	The GS115 WT strain with GM-CSF expression cassette	This study
Glyco4 CSF	The Glyco4 strain with GM-CSF expression cassette	This study
Glyco5 CSF	The Glyco5 strain with GM-CSF expression cassette	This study
GS115 ΔPp*spi1*	Pp*SPI1* gene knocked out in the GS115 WT strain	This study
GS115 ΔPp*spi1* S	Complementation of PpSpi1 in the GS115ΔPp*spi1* strain at the neutral site IV-9	This study
GS115 ΔPp*spi1* MS1	Complementation of PpSpi1 $^{(N21D,\ N51D)}$ in the GS115ΔPp*spi1* strain at the neutral site IV-9	This study
GS115 ΔPp*spi1* GS	Complementation of GFP-PpSpi1 in the GS115ΔPp*spi1* strain at the neutral site IV-9	This study
GS115ΔPp*spi1* GMS1	Complementation of GFP-PpSpi1 $^{\mathbf{(N21D,\ N51D)}}$ in the GS115ΔPp*spi1* strain at the neutral site IV-9	This study
GS115 ΔPp*spi1* GMS2	Complementation of GFP-PpSpi1 $^{\mathbf{(G114T)}}$ in the GS115ΔPp*spi1* strain at the neutral site IV-9	This study
Glyco4 GMS1	The Glyco4 strain with GFP- PpSpi1 $^{\mathbf{(N21D,\ N51D)}}$ expression cassette which inserted in the neutral site IV-9	This study
Glyco5	Overexpression of PpSpi1 in Glyco4 , the PpSpi1 expression cassette is inserted in the neutral site IV-9	This study
GS115 ΔPp*spi1* MS3	Complementation of PpSpi1 $^{\mathbf{(T38N,\ T39N,\ T45N,\ T46N,\ T53N,\ T55N,\ T57N,\ T60N,\ T61N,\ T63N,\ T65N,\ T70Q)}}$ at the neutral site IV-9 of GS115ΔPp*spi1* strain	This study
GS115 ΔPp*spi1* MS4	Complementation of PpSpi1 $^{\mathbf{(T38N,\ T39N,\ T45N,\ T46N)}}$ at the neutral site IV-9 of GS115ΔPp*spi1* strain	This study
GS115 ΔPpspi1 MS5	Complementation of PpSpi1 $^{\mathbf{(T53N,\ T55N,\ T57N,\ T60N)}}$ at the neutral site IV-9 of GS115ΔPp*spi1* strain	This study
GS115 ΔPpspi1 MS6	Complementation of PpSpi1 $^{\mathbf{(T61N,\ T63N,\ T65N,\ T70Q)}}$ at the neutral site IV-9 of GS115ΔPp*spi1* strain	This study
GS115 ΔPpspi1 GMS3	Complementation of GFP-PpSpi1 $^{\mathbf{(T38N,\ T39N,\ T45N,\ T46N,\ T53N,\ T55N,\ T57N,\ T60N,\ T61N,\ T63N,\ T65N,\ T70Q)}}$ at the neutral site IV-9 of GS115ΔPp*spi1* strain	This study
GS115 ΔPpspi1 GMS4	Complementation of GFP-PpSpi1 $^{\mathbf{(T38N,\ T39N,\ T45N,\ T46N)}}$ at the neutral site IV-9 of GS115ΔPp*spi1* strain	This study
GS115 ΔPpspi1 GMS5	Complementation of GFP-PpSpi1 $^{\mathbf{(T53N,\ T55N,\ T57N,\ T60N)}}$ at the neutral site IV-9 of GS115ΔPp*spi1* strain	This study
GS115 ΔPpspi1 GMS6	Complementation of GFP-PpSpi1 $^{\mathbf{(T61N,\ T63N,\ T65N,\ T70Q)}}$ at the neutral site IV-9 of GS115ΔPp*spi1* strain	This study
**Vectors**		
pZ-panARS-hCas9-sgRNA	The vector was construct by removing the sgRNA targeting *ADE2* gene from Z-panARS-hCas9-sgRNA (ADE2) (1), bleomycin resistance.	This study
pZQC-*GFP*-Pp*SPI1* (Mut1)-HygR	The vector harboring the upstream and downstream homologous arms of neutral site IV-9, and $P_{Pp\mathit{SPI1}}$-Pp*SPI1* signal peptide-GFP-truncated Pp*SPI1* $^{(N21D,\ N51D)}$-$T_{Pp\mathit{SPI1}}$ cassette, hygromycin resistance.	This study
pZQC-*GFP*-Pp*SPI1* (Mut1)-KanR	The vector harboring the upstream and downstream homologous arms of neutral site IV-9, and $P_{Pp\mathit{SPI1}}$-Pp*SPI1* signal peptide-GFP-truncated Pp*SPI1* $^{(N21D,\ N51D)}$-$T_{Pp\mathit{SPI1}}$ cassette, geneticin (G418) resistance.	This study
pZQC-*GFP*-Pp*SPI1* (Mut2)-KanR	The vector harboring the upstream and downstream homologous arms of neutral site IV-9, and $P_{Pp\mathit{SPI1}}$-Pp*SPI1* signal peptide-GFP-truncated Pp*SPI1* ^{G114T} -$T_{Pp\mathit{SPI1}}$ cassette, geneticin (G418) resistance.	This study
pZQC-*GFP*-Pp*SPI1*-KanR	The vector harboring the upstream and downstream homologous arms of neutral site IV-9, and $P_{Pp\mathit{SPI1}}$-Pp*SPI1* signal peptide-GFP-truncated Pp*SPI1*-$T_{Pp\mathit{SPI1}}$ cassette, geneticin (G418) resistance.	This study
pZQC-Pp*SPI1* (Mut1)-KanR	The vector harboring the upstream and downstream homologous arms of neutral site IV-9, and $P_{Pp\mathit{SPI1}}$-Pp*SPI1* $^{(N21D,\ N51D)}$-$T_{Pp\mathit{SPI1}}$ cassette, geneticin (G418) resistance.	This study
pΔ*bmt2* sgRNA Cas9	The sgRNA (5'-GGGAAGCTTCAACGACATGG-3') targeting *BMT2* gene is inserted between two BsaI sites of the vector pZ-panARS-hCas9-sgRNA,	

	northromycin resistance.	
pΔ*mnn4-3* sgRNA Cas9	The sgRNA (5'- GTTACTGTGAAGAATATGTG-3') targeting *MNN4-3* gene is inserted between two BsaI sites of the vector pZ-panARS-hCas9-sgRNA, northromycin resistance.	
pΔ*pno1* sgRNA Cas9	The sgRNA (5'- TTCATCGTAGAAGGTTACTG-3') targeting *PNO1* gene is inserted between two BsaI sites of the vector pZ-panARS-hCas9-sgRNA, northromycin resistance.	
pIV-9 sgRNA Cas9	The sgRNA (5'-ATTATCGTTTGGGATACGAG-3') targeting neutral site IV-9 is inserted between two BsaI sites of the vector pZ-panARS-hCas9-sgRNA, northromycin resistance.	This study
pΔPp*spi1* sgRNA Cas9	The sgRNA (5'-GTTTCTACCATCTCTCACGG-3') targeting Pp*SPI1* gene is inserted between two BsaI sites of the vector pZ-panARS-hCas9-sgRNA, bleomycin resistance.	This study
pGGA-ΔPp*spi1* HR-BleR	The vector harboring the upstream and downstream homologous arms of Pp*SPI1* gene, bleomycin resistance	This study
pZQC-Pp*SPI1*-KanR	The vector harboring the upstream and downstream homologous arms of neutral site IV-9, and $P_{Pp\mathit{SPI1}}$-Pp*SPI1*-$T_{Pp\mathit{SPI1}}$ cassette, geneticin (G418) resistance.	This study
pZQC-Pp*SPI1*-HygR	The vector harboring the upstream and downstream homologous arms of neutral site IV-9, and $P_{Pp\mathit{SPI1}}$-Pp*SPI1*-$T_{Pp\mathit{SPI1}}$ cassette, hygromycin resistance.	This study
pPIC9-GM-CSF	The vector harboring $P_{Pp\mathit{AOX1}}$-MATα pro-peptide fusion GM-CSF-$T_{Pp\mathit{AOX1}}$ cassette, ampicillin resistance.	This study
pGGA	The basic cloning vector with multiple cloning site (MCS), chloramphenicol resistance	NEB
pZQC-Pp*SPI1* (Mut3)-KanR	The vector harboring the upstream and downstream homologous arms of neutral site IV-9, and $P_{Pp\mathit{SPI1}}$- Pp*SPI1* $^{(T38N, T39N, T45N, T46N, T53N, T55N, T57N, T60N, T61N, T63N, T65N, T70Q)}$ -$T_{Pp\mathit{SPI1}}$ cassette, geneticin resistance.	This study
pZQC-Pp*SPI1* (Mut4)-KanR	The vector harboring the upstream and downstream homologous arms of neutral site IV-9, and $P_{Pp\mathit{SPI1}}$-Pp*SPI1* $^{(T38N, T39N, T45N, T46N)}$ -$T_{Pp\mathit{SPI1}}$ cassette, geneticin resistance.	This study
pZQC-Pp*SPI1* (Mut5)-KanR	The vector harboring the upstream and downstream homologous arms of neutral site IV-9, and $P_{Pp\mathit{SPI1}}$-Pp*SPI1* $^{(T53N, T55N, T57N, T60N)}$ -$T_{Pp\mathit{SPI1}}$ cassette, geneticin resistance.	This study
pZQC-Pp*SPI1* (Mut6)-KanR	The vector harboring the upstream and downstream homologous arms of neutral site IV-9, and $P_{Pp\mathit{SPI1}}$-Pp*SPI1* $^{(T61N, T63N, T65N, T70Q)}$ -$T_{Pp\mathit{SPI1}}$ cassette, geneticin resistance.	This study
pZQC-GFP-Pp*SPI1* (Mut3)-KanR	The vector harboring the upstream and downstream homologous arms of neutral site IV-9, and $P_{Pp\mathit{SPI1}}$-Pp*SPI1* signal peptide-GFP-truncated Pp*SPI1* $^{(T38N, T39N, T45N, T46N, T53N, T55N, T57N, T60N, T61N, T63N, T65N, T70Q)}$-$T_{Pp\mathit{SPI1}}$ cassette, geneticin resistance.	This study
pZQC-GFP-Pp*SPI1* (Mut4)-KanR	The vector harboring the upstream and downstream homologous arms of neutral site IV-9, and $P_{Pp\mathit{SPI1}}$-Pp*SPI1* signal peptide-GFP- truncated Pp*SPI1* $^{(T38N, T39N, T45N, T46N)}$ -$T_{Pp\mathit{SPI1}}$ cassette, geneticin resistance.	This study
pZQC-GFP-Pp*SPI1* (Mut5)-KanR	The vector harboring the upstream and downstream homologous arms of neutral site IV-9, and $P_{Pp\mathit{SPI1}}$-Pp*SPI1* signal peptide-GFP- truncated Pp*SPI1* $^{(T53N, T55N, T57N, T60N)}$ -$T_{Pp\mathit{SPI1}}$ cassette, geneticin resistance.	This study
pZQC-GFP-Pp*SPI1* (Mut6)-KanR	The vector harboring the upstream and downstream homologous arms of neutral site IV-9, and $P_{Pp\mathit{SPI1}}$-Pp*SPI1* signal peptide-GFP- truncated Pp*SPI1* $^{(T61N, T63N, T65N, T70Q)}$ -$T_{Pp\mathit{SPI1}}$ cassette, geneticin resistance.	This study
pZQC2	The vector harboring the upstream and downstream homologous arms of *BMT2* gene of the Gyco4 strain, and $T_{Pp\mathit{AOX1}}$-*YEA4*-$P_{Pp\mathit{VRG4}}$-$P_{Pp\mathit{GAP}}$-Mnn9 LDS-*GNT1*-$T_{Pp\mathit{CYC1}}$ cassette, bleomycin resistance. LDS: Localization Domain Sequence.	This study
pZQC3	The vector harboring the upstream and downstream homologous arms of *MNN4-3* gene of the Gyco4 strain, and $T_{Pp\mathit{CYC1}}$-*MNSII*-Mnn2 LDS-$P_{Pp\mathit{GAP}}$-$P_{Pp\mathit{RBS8B}}$-Mnn2 LDS-*GNTII*-$T_{Pp\mathit{AOX1}}$ cassette, geneticin (G418) resistance.	This study
pZQC4	The vector harboring the upstream and downstream homologous arms of *PNO1* gene of the Gyco4 strain, and $T_{Pp\mathit{CYC1}}$-*GALTI*-*UGEI*-Mnn2 LDS-$P_{Pp\mathit{GAP}}$-$P_{\mathit{PAS\_chr3\_0916}}$-UDP-galactose transporter-$T_{Pp\mathit{AOX1}}$ cassette, northromycin resistance.	This study

Reference

1. Gu Y, Gao J, Cao M, Dong C, Lian J, Huang L, Cai J, Xu Z. 2019. Construction of a series of episomal plasmids and their application in the development of an efficient CRISPR/Cas9 system in *Pichia pastoris*. World J Microbiol Biotechnol 35:79.
